# Supplementary material for: Increased CO2 fixation enables high carbon-yield production of 3-hydroxypropionic acid in yeast
Source: Nat Commun. 2024 Feb 21;15:1591. doi: 10.1038/s41467-024-45557-9 (PMC10881976; doi:10.1038/s41467-024-45557-9)
Supplement: Supplementary file 1 — Supplementary Information [file 41467_2024_45557_MOESM1_ESM.pdf]

Increased CO_2 fixation enables high carbon-yield production of 3-hydroxypropionic acid in yeast

Qin *et al.*

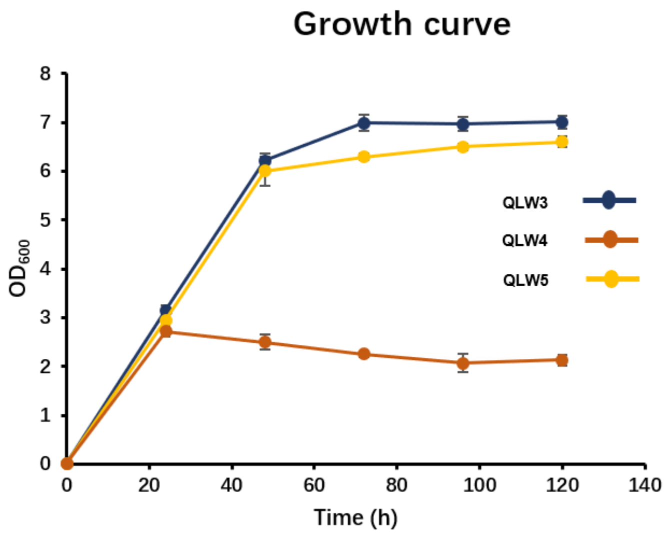

Supplementary Fig. 1. Growth profile of the QLW3, QLW4, and QLW5. The control strain was QLW3, the *STB5p::TEF1p* strain was QLW4, and the *STB5p::ARTp* strain was QLW5. The medium was defined minimal medium with 10 mM $NaHCO_3$. The genotype was listed in Supplementary Data 1. All data were presented as mean ± SD of biological triplicates. Source data are provided as a Source Data file.

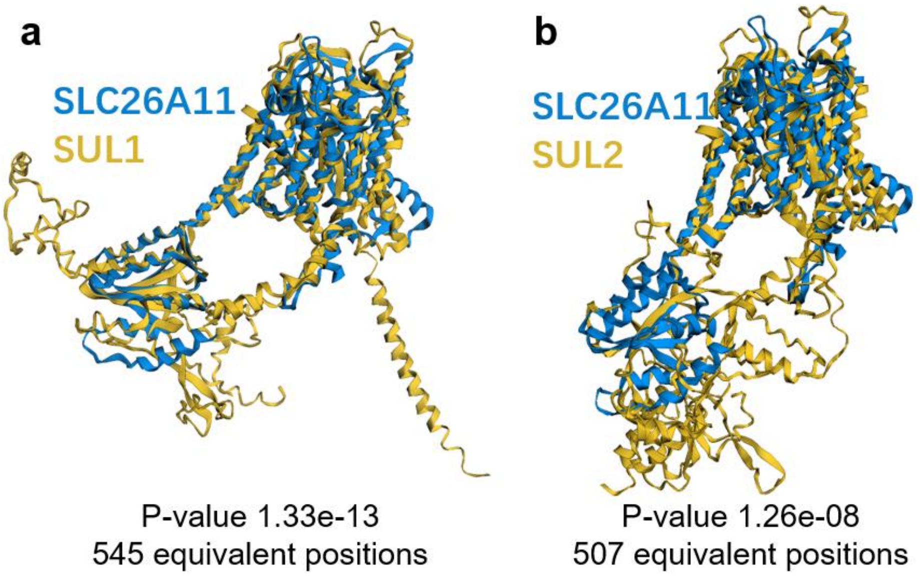

Supplementary Fig. 2. Superimposed structures of SLC26A11 with Sul1 or Sul2 . (a) Sul1; **(b)** Sul2. Protein structures for human SLC26A11 (Uniprot ID Q86WA9), yeast Sul1 (Uniprot ID P38359), and yeast Sul2 (Uniprot ID Q12325) were obtained from AlphaFoldDB (https://alphafold.ebi.ac.uk/), and structure similarity was assessed using FATCAT (https://fatcat.godziklab.org). Source data are provided as a Source Data file.

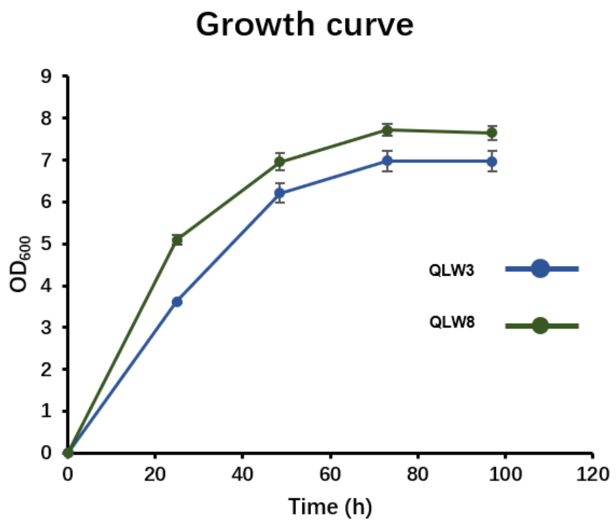

Supplementary Fig. 3. Overexpression of *SUL1* contributed to the improved strain growth. The control strain was QLW3, the *SUL1* overexpression strain was QLW8. The medium was defined minimal medium with 10 mM $NaHCO_3$. The genotype was listed in Supplementary Data 1. All data points were presented as mean ± SD of biological triplicates.

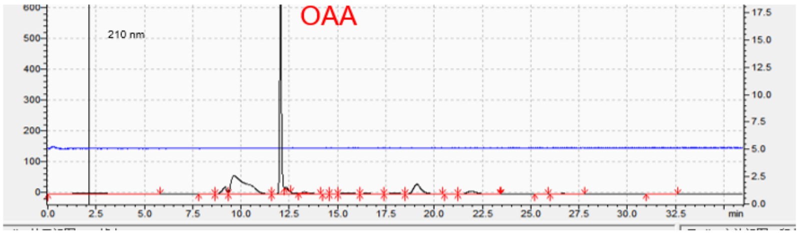

Supplementary Fig. 4. Oxaloacetate accumulation was observed after co-overexpression of *STB5* and *SUL1*. Around 10.8 minutes, there is a high accumulation peak of the oxaloacetate (OAA), the OAA detection method refer to the materials and methods section. The medium was defined minimal medium with 10 mM $NaHCO_3$.

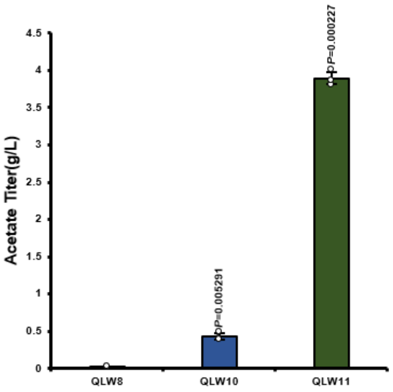

Supplementary Fig. 5. Acetate accumulation was observed after deleting the *CIT2* on QLW10. The control strain was QLW8 and QLW10, and the *CIT2* deleted strain was QLW11. The medium was defined minimal medium with 10 mM $NaHCO_3$. The genotype was listed in Supplementary Data 1. All data points were presented as mean ± SD of biological triplicates. Source data are provided as a Source Data file.

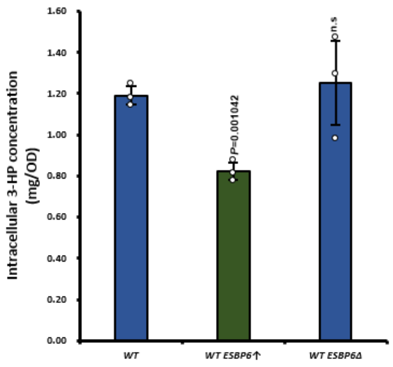

Supplementary Fig. 6. Effects of *ESBP6* gene on intracellular 3-HP concentration. The intracellular 3-HP content was measured after the treatment of strains with 50 g/L 3-HP for 24 hours to assess the impact of the *ESBP6* gene. All data points were presented as mean ± SD of biological triplicates. Source data are provided as a Source Data file.

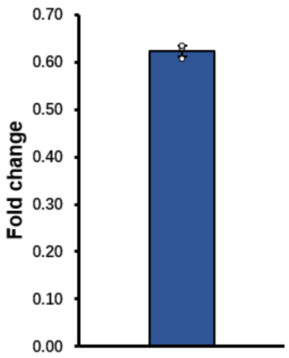

Supplementary Fig. 7. The expression level of *HIS3* marker plasmid relative to *URA3* marker plasmid. The expression level of the *HIS3* marker is approximately 62% of the *URA3* marker. The medium was defined minimal medium with 10 mM $NaHCO_3$. Abbreviations were defined in Supplementary Data 5. All data points were presented as mean ± SD of biological triplicates. Source data are provided as a Source Data file.

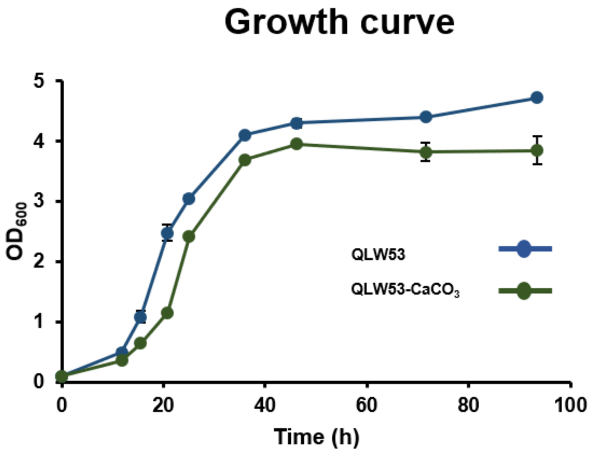

Supplementary Fig. 8. Effect of $CaCO_3$ on the QLW53 strain growth. 75 mM (0.15 g/flask) $CaCO_3$ was added to test the growth. The genotype was listed in Supplementary Data 1. All data points were presented as mean ± SD of biological triplicates. Source data are provided as a Source Data file.

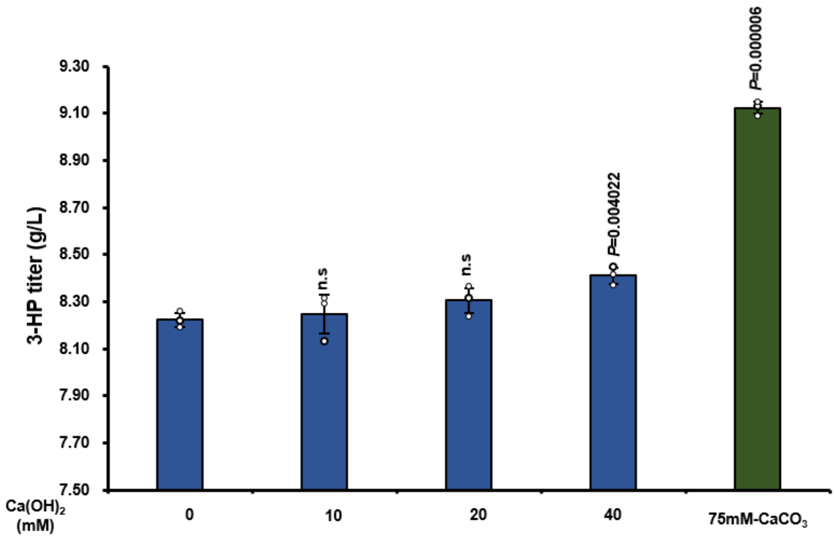

Supplementary Fig. 9. Effects of calcium hydroxide on 3-HP production. The impact of varying calcium hydroxide concentrations added to the medium on 3-HP production was investigated. 0 mM, 10 mM, 20 mM, and 40 mM represent 0 g/flask, 0.0148 g/flask, 0.0296 g/flask, and 0.0592 g/flask. All data points were presented as mean ±SD of biological triplicates. Source data are provided as a Source Data file.

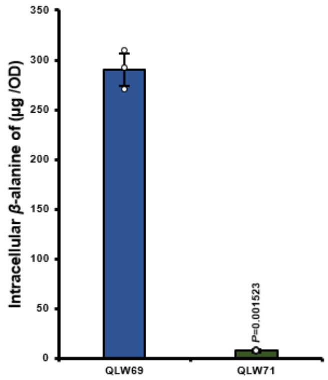

Supplementary Fig. 10. Effects of *UGA1* gene on *β*-alanine concentration in QLW69 and QLW71. The medium was defined minimal medium with 0.15 g $CaCO_3$/flask. All data points were presented as mean ± SD of biological triplicates. Source data are provided as a Source Data file.
