# Supplementary material for: Increased CO2 fixation enables high carbon-yield production of 3-hydroxypropionic acid in yeast
Source: Nat Commun. 2024 Feb 21;15:1591. doi: 10.1038/s41467-024-45557-9 (PMC10881976; doi:10.1038/s41467-024-45557-9)
Supplement: Supplementary file 3 — Description of Additional Supplementary Files [file 41467_2024_45557_MOESM3_ESM.pdf]

Description of Additional Supplementary Files

File Name: Supplementary Data 1

Description: The strain and genotype list in this paper.

File Name: Supplementary Data 2

Description: The plasmid list in this paper.

File Name: Supplementary Data 3

Description: The DNA fragment list in this paper.

File Name: Supplementary Data 4

Description: The primer list in this paper.

File Name: Supplementary Data 5

Description: The genes acronym list in this paper.

File Name: Supplementary Data 6

Description: The metabolite acronym list in this paper.
